# Localization of SARS-CoV-2 Capping Enzymes Revealed by an Antibody against the nsp10 Subunit

**DOI:** 10.3390/v13081487

**Published:** 2021-07-29

**Authors:** Vladimira Horova, Barbora Landova, Jan Hodek, Karel Chalupsky, Petra Krafcikova, Dominika Chalupska, Vojtech Duchoslav, Jan Weber, Evzen Boura, Martin Klima

**Affiliations:** Institute of Organic Chemistry and Biochemistry, Academy of Sciences of the Czech Republic, v.v.i, Flemingovo nám. 2, 166 10 Prague 6, Czech Republic; horova@uochb.cas.cz (V.H.); landova@uochb.cas.cz (B.L.); jan.hodek@uochb.cas.cz (J.H.); karel.chalupsky@uochb.cas.cz (K.C.); krafcikova@uochb.cas.cz (P.K.); chalupska@uochb.cas.cz (D.C.); duchoslav@uochb.cas.cz (V.D.); jan.weber@uochb.cas.cz (J.W.)

**Keywords:** coronavirus, SARS-CoV-2, methyltransferase, nsp10, nsp16, nsp14, capping enzyme

## Abstract

Severe acute respiratory syndrome coronavirus 2 (SARS-CoV-2) is the causative agent of the coronavirus disease-19 pandemic. One of the key components of the coronavirus replication complex are the RNA methyltransferases (MTases), RNA-modifying enzymes crucial for RNA cap formation. Recently, the structure of the 2’-*O* MTase has become available; however, its biological characterization within the infected cells remains largely elusive. Here, we report a novel monoclonal antibody directed against the SARS-CoV-2 non-structural protein nsp10, a subunit of both the 2’-*O* RNA and N7 MTase protein complexes. Using this antibody, we investigated the subcellular localization of the SARS-CoV-2 MTases in cells infected with the SARS-CoV-2.

## 1. Introduction

Antibodies are usually heterooligomeric glycoproteins that represent the most important components of the humoral part of the adaptive immune system. They are important for neutralization of pathogens such as viruses, bacteria, parasites or fungi by interfering with the pathogen attachment to the host cell. They can also activate the complement cascade, causing the lysis of pathogen cells or targeting them for internalization by phagocytic cells. The internalization and degradation of pathogens, which are opsonized or agglutinated by antibodies, by the action of phagocytes is an important mechanism for pathogen clearance. Successful recognition of target pathogen epitopes by membrane-bound antibodies can lead to the differentiation of host B lymphocytes into memory B cells and to the establishment of long-lasting immunity [1,2].

Antibodies present powerful research tools used in many common laboratory assays, such as immunofluorescence, immunoblotting, immunoprecipitation, enzyme-linked immunosorbent assays or fluorescence-activated cell sorting. They are also successful diagnostic and therapeutic tools in medical applications including fighting the coronavirus disease-19 (COVID-19) pandemic caused by the severe acute respiratory syndrome coronavirus-2 (SARS-CoV-2) [3,4]. For instance, the COVID-19 diagnostic antigen tests are usually based on antibodies against the nucleoprotein of SARS-CoV-2. Moreover, specific monoclonal antibodies that neutralize SARS-CoV-2 by targeting its spike protein can be used for the prevention and treatment of COVID-19, such as two of the FDA-approved COVID-19 drugs: LY-CoV555 (bamlanivimab) [5] and REGEN-COV (a cocktail of two monoclonal antibodies, casirivimab and imdevimab) [6]. Finally, the action of antibodies is important for several other tools for the prevention or treatment of COVID-19, such as COVID-19 vaccines or convalescent sera from patients recovered from the COVID-19 disease.

Key components of the coronavirus replication complex are the RNA-dependent RNA polymerase, helicase, nuclease and two RNA methyltransferases (MTases) [7]. Inhibitors of these enzymes are being actively developed to discover effective drugs [8,9]. The coronaviral MTases are heterodimeric protein complexes. The 2’-*O* MTase consists of the non-structural proteins nsp10 and nsp16 [10,11,12], while the N7 MTase consists of nsp10 and nsp14 [13,14]. The N7 MTase methylates the Gppp-RNA to create a cap-0 modified RNA. Subsequently, the 2’-*O* MTase methylates the 5’ end of the nascent RNA at the 2’-*O* position of its ribose ring, creating cap-1 modified RNA. This process ensures RNA stability and its efficient translation [15]. Most components of the SARS-CoV-2 replication complex were already structurally and functionally characterized in vitro [16,17,18]. Recently, the structural and biochemical analyses of the SARS-CoV-2 2’-*O* MTase have become available [19,20,21], while the structures of the coronaviral N7-MTase are available only from the SARS-CoV virus [13,14]. However, these enzymes have never been characterized in detail in the SARS-CoV-2-infected cells.

In this study, we generated a mouse monoclonal antibody against the SARS-CoV-2 nsp10 protein, a subunit of both 2’-*O* and N7 MTases. We show that the antibody specifically recognizes the nsp10 subunit both in its native conformation and in its denatured form. Using this novel antibody, we investigated the cellular localization of nsp10 during cell culture infection with the SARS-CoV-2 virus. We show that the nsp10 protein is localized mainly in vesicular structures in the perinuclear region of the infected cells, where the virus is replicated.

## 2. Materials and Methods

### 2.1. Plasmids

The SARS-CoV-2 nsp10 and nsp16 protein-encoding sequences were generated synthetically by the GeneArt synthesis (Thermo Fisher Scientific, Waltham, MA, USA). For expression of the EGFP-fused nsp10 protein in human cells, the nsp10-encoding region was cloned into *BglII* and *PstI* restriction sites of the pEGFP-C1 vector (Clontech, Mountain View, CA, USA) by restriction endonuclease recognition site cloning. For expression of the nsp10 and nsp16 proteins in *E. coli*, the nsp10- and nsp16-encoding regions were cloned into pSUMO vector containing an N-terminal His_8_-SUMO tag. All DNA constructs were verified by sequencing.

### 2.2. Protein Expression and Purification

The nsp10/nsp16 complex was expressed and purified using our standard protocols as described previously [19]. Briefly, *E. coli* BL21 DE3 cells were transformed with the expression vector and grown at 37 °C in the LB medium supplemented with 25 µM ZnSO_4_. At OD_600_ of 0.5, the protein expression was induced by 300 µM IPTG and the protein was expressed overnight at 18 °C. Bacterial cells were harvested and lysed by sonication in the lysis buffer (50 mM Tris pH 8, 300 mM NaCl, 5 mM MgSO_4_, 20 mM imidazole, 10% glycerol, 3 mM β-mercaptoethanol). The lysate was precleared by centrifugation and incubated with the HisPur Ni-NTA Superflow agarose (Thermo Fisher Scientific), and the bound proteins were extensively washed with the lysis buffer. The protein was eluted with the lysis buffer supplemented with 300 mM imidazole, dialyzed against the lysis buffer and digested with the Ulp1 protease at 4 °C overnight. The cleaved SUMO tag was removed by another incubation with the NiNTA agarose. Finally, the proteins were purified using the size exclusion chromatography at HiLoad 16/600 Superdex 200 prep grade column (GE Healthcare, Chicago, IL, USA) in the storage buffer (10 mM Tris pH 7.4, 150 mM NaCl, 5% glycerol, 1 mM TCEP).

### 2.3. Mice Immunization

All animal studies were ethically reviewed and performed in accordance with European directive 2010/63/EU and were approved by the Czech Central Commission for Animal Welfare. Female BALB/c mice were immunized on day 0 with a subcutaneous injection of 100 μg protein in complete Freud’s adjuvant (Sigma-Aldrich, St. Louis, MO, USA) (100 µL protein + 100 µL adjuvant), and on days 21, 42 and 62 with an intraperitoneal injection of 50 μg (100 µL protein + 100 µL adjuvant) protein in incomplete Freud’s adjuvant (Sigma-Aldrich). Spleens were harvested on day 64. Anti-nsp10/nsp16 antibodies-producing mouse splenocytes were fused with myeloma cells and the candidate hybridomas were selected using the commercial service of the Monoclonal Antibodies and Cryobank facility at the Institute of Molecular Genetics of the Czech Academy of Sciences.

### 2.4. Tissue Culture, Transfections and SARS-CoV-2 Infection

Human cervical carcinoma cells HeLa and monkey kidney epithelial cells Vero-E6 were maintained in Dulbecco’s modified Eagle’s medium (DMEM; Sigma-Aldrich) supplemented with 10% fetal bovine serum (FBS; Thermo Fisher Scientific/Gibco, Waltham, MA, USA). For transfection, HeLa cells were plated onto a 4-chamber 35 mm dish with a glass bottom (Cellvis, Mountain View, CA, USA). Plasmid DNA was transfected with X-tremeGENE HP DNA Transfection Reagent (Sigma-Aldrich) according to the manufacturer’s protocol. For SARS-CoV-2 infection, one day prior to infection, Vero-E6 cells were seeded in a 4-chamber 35 mm dish with a glass bottom (Cellvis) at 180,000 cells per chamber. The next day, cells were infected with the SARS-CoV-2 strain *hCoV-19/Czech Republic/NRL_6632_2/2020* in our BSL3 facility at MOI 0.5 in DMEM medium supplemented with 2% FBS and incubated at 37 °C in the CO_2_ incubator for 24–72 h.

### 2.5. Enzyme-Linked Immunosorbent Assay (ELISA)

The recombinant SARS-CoV-2 nsp10 protein was used to coat the wells of the microtiter Maxisorp Nunc-Immuno plates at 2 μg/mL in the coating buffer (30 mM Na_2_CO_3_, 70 mM NaHCO_3_, pH 9.5) at 4 °C overnight. Wells were washed with phosphate-buffered saline (PBS) supplemented with 0.05% Tween-20 between every following step. Wells were blocked with PBS with 2% milk for 2 h at room temperature with gentle shaking. Then, supernatants from selected hybridomas were added in a series of dilutions and incubated for 2 h at room temperature with gentle shaking. Afterwards, the HRP-conjugated goat anti-mouse antibody (diluted 1:5000 in PBS with 0.2% milk) was added and incubated for 1 h at room temperature with gentle shaking. The colorimetric reaction was performed with the chromogenic TMB substrate added for 30 min without shaking. The reaction was stopped with 1 M H_2_SO_4_ and the absorbance at 450 nm was determined using a Tecan plate reader.

### 2.6. Immunofluorescence Assay

At 24 h post transfection or 24–72 h post SARS-CoV-2 infection, cells were washed with PBS, fixed with 4% paraformaldehyde in PBS for 15 min at room temperature and permeabilized with 0.2% Triton X-100 in PBS for 5 min. Fixed samples were blocked with 1% bovine serum albumin in PBS for 1 h and immunostained with the appropriate primary and secondary antibodies diluted in DMEM supplemented with 10% FBS. Sources of the antibodies were as follows: mouse antibody anti-nsp10 (described here), rabbit antibody anti-golgin-97 (D8P2K; Cell Signaling Technology, Danvers, MA, USA), anti-PDI (C81H6; Cell Signaling Technology), anti-giantin (BLD-924302; BioLegend, San Diego, CA, USA), anti-dsRNA (ABA-AB00458-23.0; Biozol, Eching, Germany), anti-SARS-CoV-2 spike protein (40150-R007; SinoBiological, Beijing, China), and goat-anti-mouse and goat-anti-rabbit secondary antibodies conjugated to Alexa Fluor 647 and 488, respectively (Thermo Fisher Scientific). Nuclei were stained with Hoechst 33342 (Thermo Fisher Scientific) for 1 min, followed by the final wash with PBS, and then the samples were directly imaged in PBS. All images were acquired on a Zeiss LSM 780 confocal microscope running ZEN 2.3 SP1 (black) software, using a 40×/1.2 water objective. The Zen 2.3 software (blue edition) was used for image processing, microscopic data analysis and creation of two-color fluorescence intensity profiles. The Fiji image processing package [22] and JaCoP plug-in [23] were used to calculate the Pearson correlation coefficient.

## 3. Results

**Figure 1:**
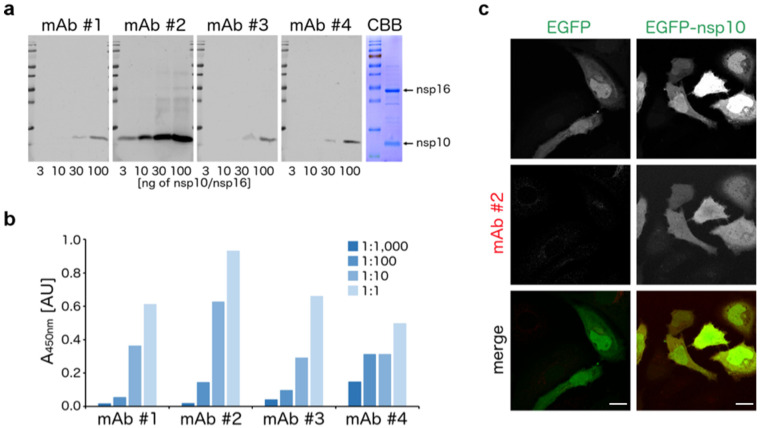
Specific interaction of the anti-nsp10 monoclonal antibody with both denatured and native nsp10 protein. (**a**) Indicated amounts of the recombinant SARS-CoV-2 nsp10/nsp16 complex were visualized either by SDS-PAGE coupled with Coomassie Brilliant Blue (CBB) staining, or by Western blotting coupled with immunostaining with supernatants from four candidate anti-nsp10/nsp16 antibodies-producing hybridomas. (**b**) Recombinant SARS-CoV-2 nsp10 protein was measured by the enzyme-linked immunosorbent assay (ELISA) using the indicated series of dilutions of supernatants from four candidate anti-nsp10/nsp16 antibodies-producing hybridomas. (**c**) EGFP-fused SARS-CoV-2 nsp10 protein or EGFP alone as a control were overexpressed in HeLa cells by transient transfection. The cells were fixed and immunostained with the supernatant from the selected hybridoma. Expression of nsp10 (red) and EGFP (green) was analyzed by confocal microscopy. Scale bars represent 20 µm.

We immunized mice with the native recombinant SARS-CoV-2 nsp10/nsp16 complex and using standard techniques (detailed in the Section 2), we generated and selected four hybridomas producing anti-nsp10/nsp16 antibodies. Since the mice immunization and selection of candidate hybridomas were performed using a native protein complex, we investigated whether our antibodies were also capable of specifically recognizing the denatured antigen. We subjected the recombinant SARS-CoV-2 nsp10/nsp16 complex to denaturing SDS-PAGE and analyzed it by immunoblotting using supernatants from four selected hybridomas. We discovered that all four supernatants specifically interacted with a band corresponding to the SARS-CoV-2 nsp10 protein; however, the nsp16 subunit was not recognized by any antibody (Figure 1a). One supernatant (sample no. 2 in Figure 1a) recognized the nsp10 protein in the low nanogram range.

The specificity of these supernatants towards the nsp10 protein and their ability to interact with the protein in its native conformation was confirmed by the enzyme-linked immunosorbent assay (ELISA). In this assay, all four supernatants recognized the recombinant SARS-CoV-2 nsp10 protein in a dose-dependent manner (Figure 1b). The highest response was achieved again with the supernatant no. 2, which was therefore chosen for further immunocytochemistry experiments. HeLa cells were transfected with EGFP-fused SARS-CoV-2 nsp10 or EGFP alone as a control, immunostained with this anti-nsp10 antibody and imaged using confocal microscopy. The signal of the antibody was present only in cells transfected with EGFP-fused nsp10, but not in cells transfected with EGFP alone, thereby confirming the specificity of the anti-nsp10 antibody (Figure 1c).

**Figure 2:**
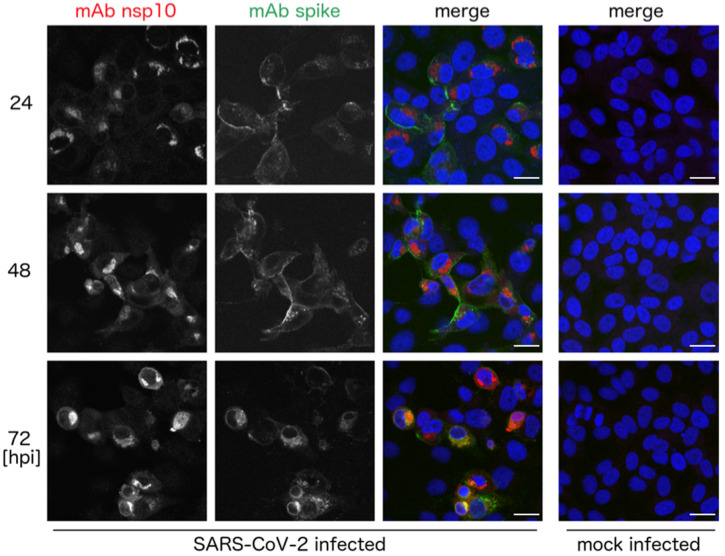
Expression of the nsp10 and spike proteins in the SARS-CoV-2-infected cells. Vero cells were infected with the SARS-CoV-2 strain *hCoV-19/Czech Republic/NRL_6632_2/2020*, fixed at indicated times and immunostained with anti-nsp10 and anti-spike antibodies. Expression of nsp10 (red) and spike (green) proteins was analyzed by confocal microscopy. Nuclei were stained with Hoechst 33342 (blue). Scale bars represent 20 µm. Hpi, hours post infection.

Once the specificity of the antibody was verified, we investigated whether it was sensitive enough to detect the SARS-CoV-2 nsp10 protein in cells infected with SARS-CoV-2. We infected Vero cells with SARS-CoV-2 and used the antibody to monitor the expression and subcellular localization of the nsp10 protein by confocal microscopy. During a time course of 1–3 days post infection, we observed a clear signal of the nsp10 protein localized in the perinuclear region of infected cells. As expected, the nsp10 signal was observed in cells where we could detect expression of the SARS-CoV-2 spike protein taking advantage of a commercially available anti-spike antibody (Figure 2).

**Figure 3:**
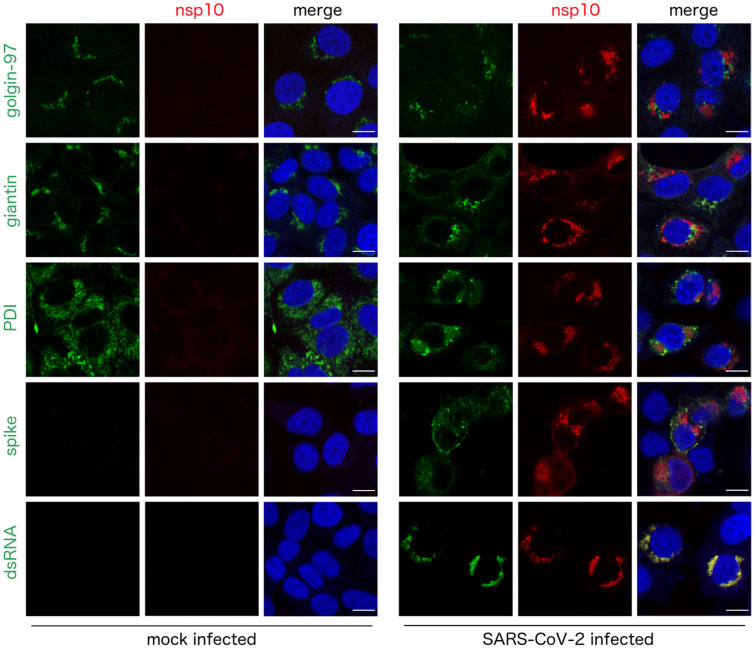
Subcellular localization of the nsp10 protein in the SARS-CoV-2-infected cells. Vero cells were infected with the SARS-CoV-2 strain *hCoV-19/Czech Republic/NRL_6632_2/2020*, fixed at 36 h post infection and immunostained with antibodies as indicated. Subcellular localization of the nsp10 protein (red) and its co-localization with PDI (a marker of endoplasmic reticulum), giantin, golgin-97 (markers of Golgi), spike protein of SARS-CoV-2 and dsRNA (a marker of viral replication; green) were analyzed by confocal microscopy. Nuclei were stained with Hoechst 33342 (blue). Scale bars represent 10 µm.

**Figure 4:**
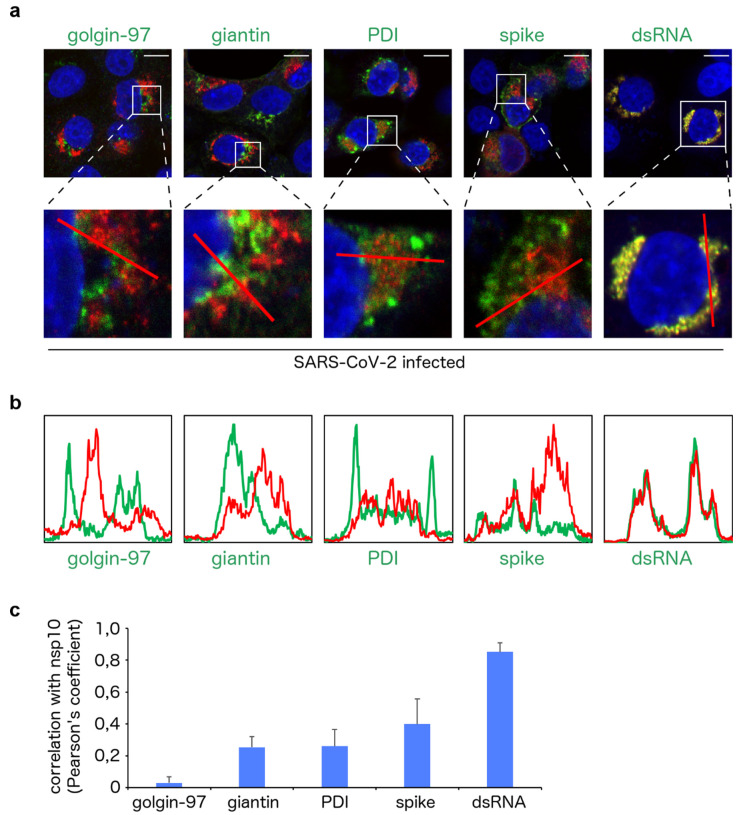
Analysis of the nsp10 subcellular localization in the SARS-CoV-2-infected cells. (**a**) A detailed view of the subcellular localization of the nsp10 protein (red) and its co-localization with several markers (green) as in Figure 3. Scale bars in the upper panel represent 10 µm. (**b**) Co-localization of the nsp10 protein (red) with several markers (green) is presented as the fluorescent intensity profile plots. The plots were generated using the selections indicated by the red lines in the magnified images in the bottom panel of Figure 4a. (**c**) Statistical analysis of the co-localization of the nsp10 protein with several markers is presented as Pearson correlation coefficients ± standard deviations from at least 10 cells.

Next, we analyzed the localization of the nsp10 protein in greater detail. Vero cells were infected with SARS-CoV-2 again and the localization of the nsp10 protein and several markers, such as markers of the endoplasmic reticulum (ER) and Golgi, at 36 h post infection was visualized (Figure 3). Localization of ER and Golgi markers slightly changed in infected cells compared to uninfected controls, documenting membrane remodeling upon coronaviral infection (Figure 3). Co-localization of the nsp10 protein with the markers was analyzed using the fluorescent intensity profile plots (Figure 4a,b) and calculation of the Pearson correlation coefficients (Figure 4c). As expected, given the function of the nsp10 protein in RNA replication, we observed its highly significant co-localization with the double-stranded RNA (dsRNA), a hallmark of the sites of the SARS-CoV-2 virus replication (Figure 3 and Figure 4). In contrast to dsRNA, we observed only a little to insignificant co-localization of the nsp10 protein with the protein disulfide isomerase (PDI), a marker of ER, or with giantin and golgin-97, markers of the Golgi (Figure 3 and Figure 4).

In conclusion, we generated a mouse monoclonal antibody specific to the SARS-CoV-2 nsp10 protein, a subunit of the coronaviral 2’-*O* and N7 MTases. It recognizes both the denatured protein and the native protein expressed in either transfected or SARS-CoV-2 infected cells. The antibody revealed that within infected cells, the nsp10 protein is localized in specific vesicular structures in the perinuclear region of the cell where the virus replicates, yet is distinct from the ER or the Golgi system.

## 4. Discussion

Positive-sense single-stranded RNA (+RNA) viruses such as coronaviruses replicate at specific membranous compartments known as replication organelles (ROs). Membranes of the viral ROs arise from membranes of the host cell and serve as platforms for the assembly of the viral replication complexes. In general, +RNA viruses can hijack almost any host membranes, such as the ER, Golgi system, trans-Golgi network, endosomal, plasma or the outer mitochondrial membranes [24,25,26,27,28,29]. It has been reported that viruses across various coronavirus genera induce a similar type of membrane structures. Their ROs consist of double-membrane vesicles (DMVs) and convoluted membranes, which form an interconnected reticulo-vesicular network of remodeled membranes connected with ER in the perinuclear region [30,31]. The interior of DMVs can be interconnected with the cytoplasm through a specific molecular pore complex, possibly allowing RNA exchange [32]. Formation of DMVs upon coronavirus infection is predominantly driven by the viral non-structural proteins nsp3, nsp4 and nsp6 [33,34]. DMVs represent the main sites of coronaviral RNA synthesis across various coronavirus species [35] and recently, they have been reported as the main type of the SARS-CoV-2 ROs as well [36,37]. During SARS-CoV-2 infection, biogenesis of DMVs is accompanied by other events such as the extensive Golgi fragmentation, alteration of mitochondrial network, recruitment of peroxisomes to the viral ROs, profound remodeling of cytoskeleton elements, virion assembly and budding events [37].

In this study, we generated a novel monoclonal antibody against the SARS-CoV-2 nsp10 protein, a subunit of the coronaviral RNA-capping complex, and used it as a tool to monitor subcellular localization of the nsp10 protein during viral infection. As expected, the nsp10 protein was localized in the vesicular compartments in the perinuclear region of the infected cells, which co-localized with the sites of viral replication monitored by the presence of dsRNA. DsRNA, an intermediate of viral replication, has been reported to segregate into the interior of DMVs during both SARS-CoV and SARS-CoV-2 infection [30,38,39].

In agreement with previous studies describing formation of the SARS-CoV-2 ROs in the perinuclear region close to ER and Golgi, we observed a proximity but not a co-localization of nsp10 with several ER (PDI) and Golgi (giantin, golgin-97) markers. An altered pattern of these markers in infected cells compared to uninfected controls indicated that the SARS-CoV-2 infection caused a rearrangement of the ER and Golgi compartments in the host cell. We also showed that the SARS-CoV-2 nsp10 protein only poorly co-localized with the spike protein. In contrast to nsp10, which is localized mainly in the perinuclear region close to ER and Golgi, the spike protein was present especially on the surface of the infected cells. These findings correspond to the described SARS-CoV-2 life cycle analyzed by cryo-electron tomography and transmission electron microscopy [35,36,38]. These studies confirm that early viral RNA replication is located in DMVs in the perinuclear region, whereas virion assembly and budding occurs mainly in the ER–Golgi intermediate compartment (ERGIC) and, due to exocytosis, at the plasma membrane.

## Data Availability

Not applicable.
